# Modulation of Motor Cortical Activities by Action Observation and Execution in Patients with Stroke: An MEG Study

**DOI:** 10.1155/2019/8481371

**Published:** 2019-10-30

**Authors:** Jun-Ding Zhu, Chia-Hsiung Cheng, Yi-Jhan Tseng, Chien-Chen Chou, Chih-Chi Chen, Yu-Wei Hsieh, Yu-Hsien Liao

**Affiliations:** ^1^Department of Occupational Therapy and Graduate Institute of Behavioral Sciences, College of Medicine, Chang Gung University, Taoyuan, Taiwan; ^2^Healthy Aging Research Center, Chang Gung University, Taoyuan, Taiwan; ^3^Department of Psychiatry, Chang Gung Memorial Hospital, Linkou, Taiwan; ^4^Department of Medical Research, Hsinchu MacKay Memorial Hospital, Hsinchu, Taiwan; ^5^Epilepsy Division, Neurological Institute, Taipei Veterans General Hospital, Taipei, Taiwan; ^6^Department of Physical Medicine and Rehabilitation, Chang Gung Memorial Hospital, Linkou, Taiwan; ^7^School of Medicine, College of Medicine, Chang Gung University, Taoyuan, Taiwan; ^8^Department of Physical Medicine and Rehabilitation, Chang Gung Memorial Hospital, Taoyuan, Taiwan

## Abstract

Action observation therapy has recently attracted increasing attention; however, the mechanisms through which action observation and execution (AOE) modulate neural activity in stroke patients remain unclear. This study was aimed at investigating the effects of action observation and two types of AOE on motor cortical activations after stroke using magnetoencephalography. Twenty patients with stroke and 20 healthy controls were recruited for the collection of data on the beta oscillatory activity in the primary motor cortex (M1). All participants performed the conditions of resting, observation only, and video observation combined with execution (video AOE). Stroke patients performed one additional condition of affected hand observation combined with execution (affected hand AOE). The relative change index of beta oscillations was calculated, and nonparametric tests were used to examine the differences in conditions. In stroke patients, the relative change index of M1 beta oscillatory activity under the video AOE condition was significantly lower than that under the observation only and affected hand AOE conditions. Moreover, M1 cortical activity did not significantly differ under the observation only and affected hand AOE conditions. For healthy controls, the relative change index under the video AOE condition was significantly lower than that under the observation only condition. In addition, no significant differences in relative change indices were found under the observation only and video AOE conditions between the 2 groups. This study provides new insight into the neural mechanisms underlying AOE, which supports the use of observing videos of normal movements during action observation therapy in stroke rehabilitation.

## 1. Introduction

Stroke is the leading cause of long-term disability in adults worldwide [1, 2]. A large proportion of patients with stroke experience upper extremity motor deficits that contribute to their functional disability [3]. Given the debilitating effects of stroke on motor skills, neurorehabilitation researchers and practitioners have focused on developing and providing effective motor rehabilitation to patients with stroke [4]. Recently, the use of action observation therapy to enhance motor-skill learning and to promote neural plasticity changes in patients with stroke has received increased attention [5–11].

Action observation is defined as “a dynamic state during which an observer can understand what other people are doing by simulating the actions and the outcomes that are likely to follow from the observed motor act” [9, 12]. It is a type of augmentation of a motor-based technique with cognitive strategies. The neurophysiological basis of action observation involves the mirror neuron system and action observation network. In these networks, the same neural areas are responsible for both an observed action performed by others and the actual execution of the action [5, 13–15]. Action observation is applied in stroke rehabilitation because it may prime the motor system for subsequent motor practice and enhance the patients' performance by activating these networks [5, 16–18].

Action observation therapy commonly includes two phases: action observation and action execution. In the observation phase, patients are required to carefully observe movements or daily actions performed by others as if the patients themselves are performing these actions [5]. This phase aims to restore the neural structures that are normally recruited during the execution of the observed actions. Experimental studies involving humans have shown that an action observation-execution matching mechanism is present in specific regions of the frontal and parietal lobes [19, 20]. However, many imaging studies on action observation have been conducted on healthy controls [14, 16, 19–21]. The experimental studies involving stroke patients have had considerable disparities among the stroke-onset phases (subacute or chronic stroke) of the recruited patients and the sizes (5 to 24) of the study samples. These studies also applied different neuroimaging tools (TMS, fMRI, or EEG) and observed different actions (transitive or intransitive movements) [8, 11, 22–28]. More importantly, many of the studies only investigated the effects of action observation or observation with imagery on neural activations; they did not incorporate action observation with action execution during the imaging experiments in patients with stroke [8, 11, 23–25, 27]. The evidence on the neural mechanisms during action observation and execution remains limited and inconclusive. However, action observation with execution (i.e., physical practice) has been more commonly applied in the clinical practice of stroke rehabilitation than has only observing an action without physically executing it. There is a gap between the imaging study designs and clinical practice of this therapy, so more studies to unravel the responses of neural activities to action observation and execution in patients with stroke are required.

In addition, patients with stroke commonly observe their awkward upper extremity (i.e., affected side) while performing reaching/grasping movements or executing functional tasks in clinical and practical settings [29]. Recent magnetoencephalography (MEG) studies have found that in healthy adults, the observation of normal hand movements produces stronger activations and greater functional connectivity in the motor and somatosensory cortex than does the observation of abnormal movements [29, 30]. However, no MEG studies that directly compared the neural activities exhibited by patients with stroke when observing normal versus distorted, abnormal hand movement patterns have been conducted. The neural mechanisms involved when stroke patients observe videos of movements performed by a healthy adult (i.e., normal movements) versus when they observe their own awkward hand movements (i.e., abnormal movements), as commonly performed in clinical situations, warrant further investigation. This study is the first to investigate this issue in stroke patients so as to provide neurophysiological findings to inform clinical rehabilitation practice.

This study is aimed at applying MEG to demonstrate the modulation of corticomotor activity by action observation and execution (AOE). We designed 3 experimental conditions to comprehensively compare the effects of AOE. The 2 study hypotheses were the following (1): neural activity in the primary motor cortex (M1) would be stronger under the condition of video observation combined with execution (i.e., video AOE) than under the observation only condition in stroke patients and healthy controls and (2) M1 corticomotor activity would be more pronounced under the video AOE condition than under the affected hand observation combined with execution (i.e., affected hand AOE) condition in stroke patients.

## 2. Materials and Methods

### 2.1. Participants

Twenty patients with stroke were recruited (Table 1). The average age of the stroke patients was 48.55 ± 8.89 years. There were 11 patients with ischemic stroke and 9 patients with hemorrhagic stroke. Fourteen patients had right hemispheric lesions, and 6 patients had left hemispheric lesions. All patients were right-handed by self-report and the Edinburgh Handedness Inventory [31]. All patients met the following inclusion criteria (1): unilateral stroke (2), 1 month to 12 months since the stroke onset (3), scores of 18 to 60 on the upper-extremity subscale of the Fugl-Meyer Assessment [32] (4), age of at least 20 years, and (5) ability to follow the study instructions. Patients were excluded on the basis of the following exclusion criteria (1): severe visual deficits or spatial neglect (2), global or receptive aphasia (3), other neurological or psychiatric disorders, and (4) presence of metal implants or other factors that might interfere with MEG recordings. The medical information of the participants, including the types of stroke and lesion sites, was reviewed by a neurologist or physiatrist according to the medical records and available brain imaging scans.

Moreover, twenty right-handed healthy controls were also enrolled in this study (mean age: 55.25 ± 9.55, 9 males). The Institutional Review Board of Taipei Veterans General Hospital approved this study, and all participants provided written informed consent.

### 2.2. Experimental Procedures

**Figure 1:**
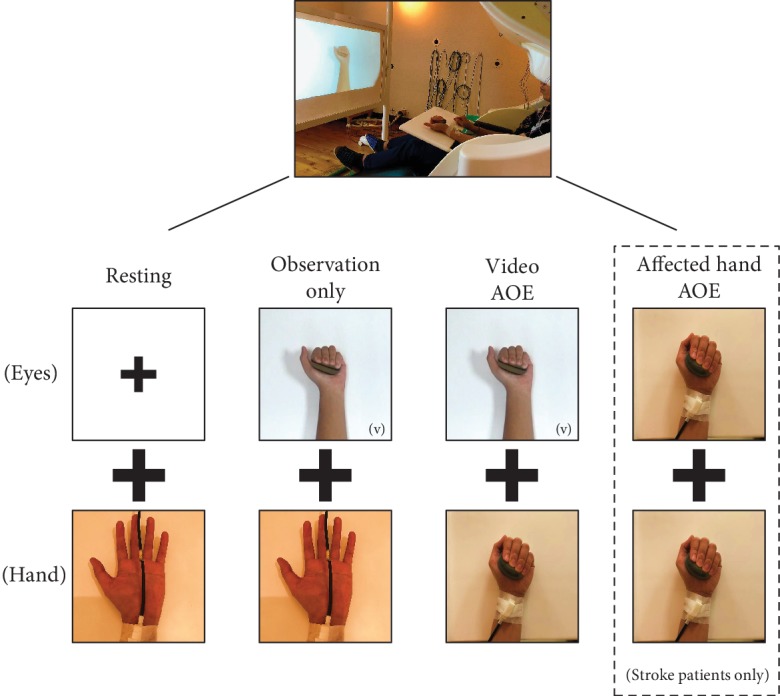
Illustration of the conditions during MEG recordings. Note: (v) in some pictures indicates watching video movements. AOE = action observation and execution.

All the experimental conditions were completed by each participant on the same day (Figure 1). In this study, the resting condition was set as a baseline reference for calculating the relative change indexes of the other experimental conditions. In the resting condition, all participants were asked to fixate on a crosshair reticle in front of them. In addition, the 3 experimental conditions included the following (1): The *observation only* condition: the stroke patients were asked to watch a video that showed a healthy actor gripping a soft ball with the right or left hand, whichever was consistent with their affected hand. The healthy controls were asked to watch a video which showed a healthy actor gripping a soft ball with the left hand (nondominant hand) (2). The video observation combined with execution (*video AOE*) condition: the stroke patients were asked to watch a video of a healthy actor gripping a soft ball and to simultaneously imitate and execute the same movement with their affected hand. The healthy controls were instructed to perform the same procedure but to execute the movement with the left hand (3). The affected hand observation combined with execution (*affected hand AOE*) condition: the stroke patients were asked to grip a soft ball with the affected hand while simultaneously observing the movement of their affected hand. This condition simulated a customary rehabilitation clinic context, in which stroke patients commonly execute tasks with their affected hand and simultaneously watch the affected hand's movements. Only stroke patients performed this condition because it was similar to a clinical situation. The healthy controls did not have an affected hand and thus did not perform this condition. Each condition lasted for 4 to 4.5 minutes. Except for the resting condition, all other conditions were performed in a counterbalanced sequence.

M1 neuromagnetic activities under the conditions were recorded with a whole-head 306-channel MEG (Vectorview, Elekta Neuromag, Helsinki, Finland). In this study, the participants comfortably sat in the MEG scanner environment with their heads fully supported by the inner posterior wall of the helmet-shaped device (Figure 1).

Electrical stimuli with 0.2 ms constant-current square-wave pulses were delivered to the median nerve of the stroke patient's affected hand or healthy participant's left hand throughout all experiment conditions. Beta oscillatory activity could be generated and detected in the M1 of the stroke patient's lesioned hemisphere or healthy control's right hemisphere in response to the electrical stimulation or finger movements [33, 34]. The strength of the beta oscillatory activity immediately decreased after median nerve stimulation and then exceeded the prestimulus baseline level in the time window of 0.4 s to 0.9 s (i.e., beta rebound) [35, 36]. An electrical stimulator was used (Konstantstrom Stimulator, Schwind, Erlangen, Germany) with a constant interstimulus interval (ISI) of 1.5 s and a stimulus intensity of 20% above the motor threshold to obtain cortical responses with a good signal-to-noise ratio [37]. During the experiments, all participants were asked to follow the instructions for each condition and to ignore the electrical stimuli [37]. Electrical stimuli were continuously presented for 4 to 4.5 minutes. During this time period, patients with stroke performed 16 to 18 repetitions of gripping and releasing movements in each condition.

### 2.3. MEG Recordings

The MEG comprised 102 identical triple sensors. Each sensor element consisted of two planar gradiometers and one magnetometer. The planar gradiometers, which could detect the largest signals directly above the activated neural regions, were analyzed. The exact locations on the head with respect to the sensors were measured by head indicator coils, and the coil locations in relation to anatomical landmarks (left preauricular point, right preauricular point, and nasion) were determined with a 3D digitizer. During MEG scanning, the patients were instructed not to move their heads and were allowed short breaks between different experimental blocks. The digitization of the MEG signals was set at a sampling rate of 1000 Hz with an online bandpass of [0.1, 120] Hz. An interval of 1100 ms, including a prestimulus baseline of 100 ms, was evaluated. The average percentage of rejected trials was 14.6% (range: 0% to 50%) due to eye blinks of the participants or synergy movements of the patients with stroke. However, at least 90 artifact-free epochs under each condition were collected for further analyses.

In addition, surface electromyography was applied to each participant. The recorders were placed on the flexor digitorum superficialis with the bandpass filter within 20–200 Hz off-line. The absolute magnitude was calculated by rectifying the filtered signals. The electromyogram signals from artifact-free epochs in each experimental condition were then averaged to record muscle activities over time [30].

### 2.4. MEG Data Analysis

To suppress environmental magnetic interference from the MEG data, the temporal signal-space separation method was employed in this study [38]. Brainstorm software [39] was utilized to obtain the modeling of M1 beta oscillations. The folded cortical surface was used to resolve the forward problem by using an overlapping sphere model [40]. The Montreal Neurological Institute (MNI) brain template (ICBM152) was applied to geometrically rescale the source map of each subject. An M1 region with a size of approximately 4–5 cm^2^ was manually identified as the region of interest [30, 41]. The reconstruction of the source activation was resolved by the depth-weighted minimum norm estimate (wMNE). To further characterize spectral responses, the Morlet wavelet-based time-frequency approach (central frequency: 1 Hz; time resolution: 3 s) was applied to transform the MEG-source waveforms from each raw trial (100 ms before and 1000 ms after the stimulus onset) after the exclusion of prominent electrooculogram artifacts by the Signal-Space Projection (SSP) method with default settings in the Brainstorm software.

The identification and calculation of the mean strength of the most reactive M1 activations, i.e., ∼20 Hz oscillations (2 Hz for consecutive bins), were based on the average of 100 ms centering peak latency (50 ms before and after the peak) [41]. A large reduction in beta oscillatory power is indicative of greater M1 activations. The time-resolved magnitude of each primary source was normalized to its fluctuations over the baseline, which was converted into *z*-score time series at each cortical location. Then, the *z*-score values were used to compute the absolute magnitude changes in each participant in accordance with the baseline levels of each condition [30, 42, 43].

A relative change index of beta oscillatory activity was calculated from the value of beta oscillatory activity in each experimental condition minus that of the resting condition divided by that of the resting condition to correct the differences in the baseline. A greater decrease in the relative change index indicates greater activations. The formula is ((experimental condition − resting condition)/resting condition) × 100%.

### 2.5. Statistical Analysis

To examine the within-group differences in stroke patients, a nonparametric Friedman test was applied to compare the relative change indices of beta oscillatory activities among the 3 experimental conditions (i.e., observation only vs. video AOE vs. affected hand AOE). For post hoc comparisons, the Wilcoxon signed-rank test with Bonferroni correction was used (*p* value < 0.0167). In healthy controls, the nonparametric Wilcoxon signed-rank test was used to examine the relative change indices between the 2 experimental conditions (i.e., observation only vs. video AOE).

To examine the group differences, the nonparametric Mann–Whitney *U* test was applied to compare the relative change indices of the observation only and video AOE conditions between the stroke patients and healthy controls. The Bonferroni correction was used for the comparisons of the 2 conditions, and a *p* value < 0.025 indicated statistical significance.

## 3. Results

**Figure 2:**
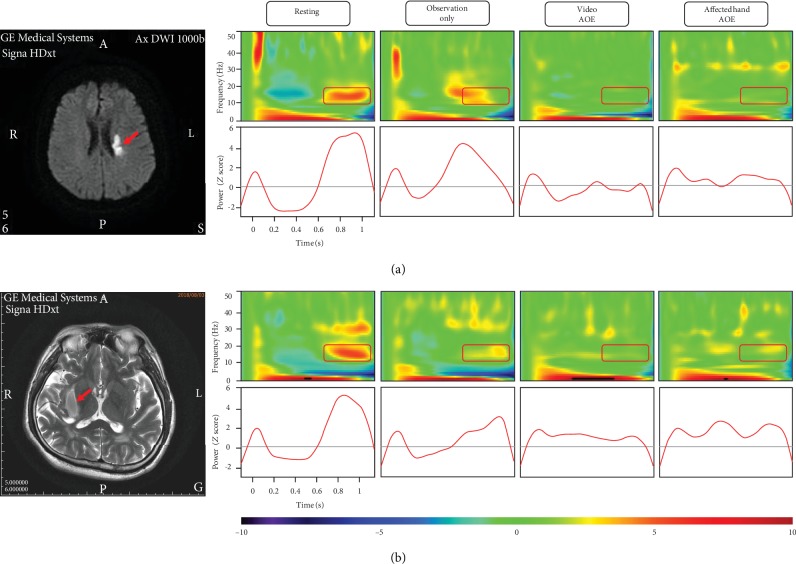
The brain images of stroke lesions and time-frequency analyses of beta oscillatory activities of 2 individual patients. (a) Patient No. 18 had an ischemic stroke in the left-side corona radiata. Left panel: the lesion site of this patient (red arrow) in the diffusion-weighted MRI image. Right panel: time-frequency maps and time courses of beta oscillatory activity under each condition. The powers of beta oscillatory activity of this patient in the resting, observation only, video AOE, and affected hand AOE conditions were 5.05, 4.54, 0.15, and 1.21, respectively. (b) Patient No. 20 had a hemorrhagic stroke in the right-side putamen. Left panel: the lesion site of this patient (red arrow) in the T2-weighted MRI image. Right panel: time-frequency maps and time courses of oscillatory activity under each condition. The powers of beta oscillatory activity of this patient in the resting, observation only, video AOE, and affected hand AOE conditions were 4.71, 2.86, 0.97, and 2.26, respectively. Note: AOE = action observation and execution.

Figure 2 illustrates the lesion sites and the time-frequency analyses of beta oscillatory activities under each MEG condition of 2 individual patients (No. 18 and No. 20) as examples to demonstrate individual responses.

**Figure 3:**
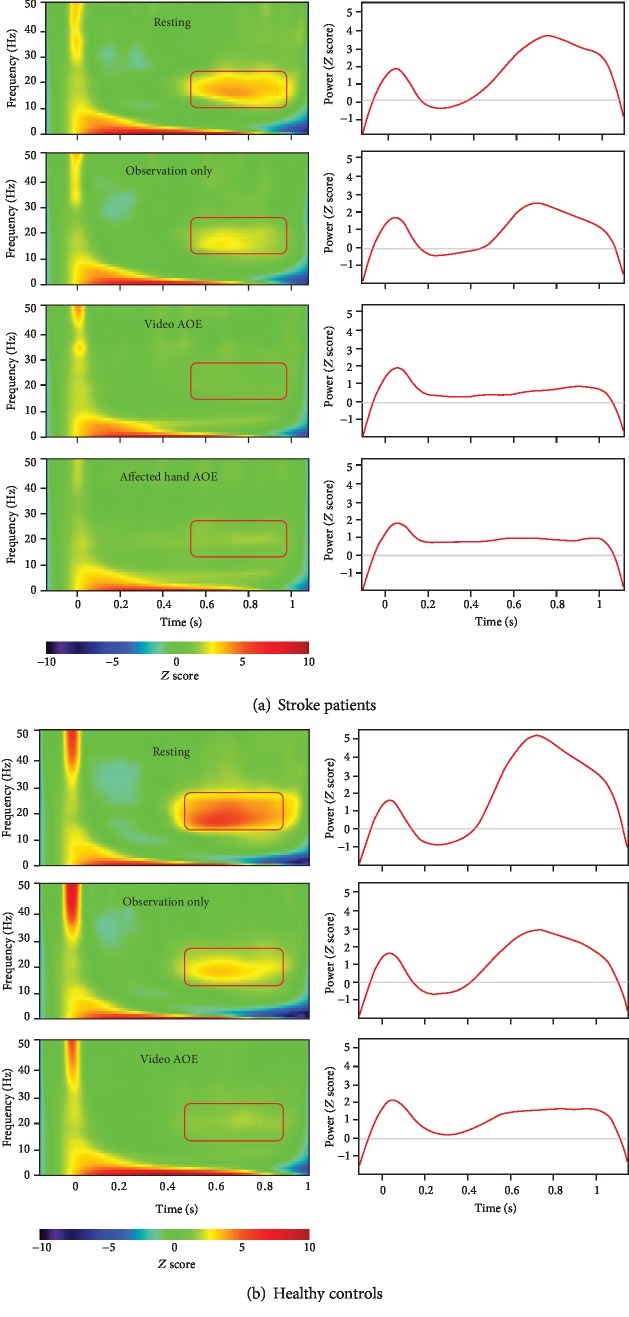
Left panel of (a, b): time-frequency maps of group-averaged electricity-induced beta rebound oscillations (red rectangles) under each condition. Right panel of (a, b): time courses of beta oscillatory activities in the most reactive frequency bands (2 Hz for consecutive bins) in the primary motor cortex (M1). Note: AOE = action observation and execution.

**Figure 4:**
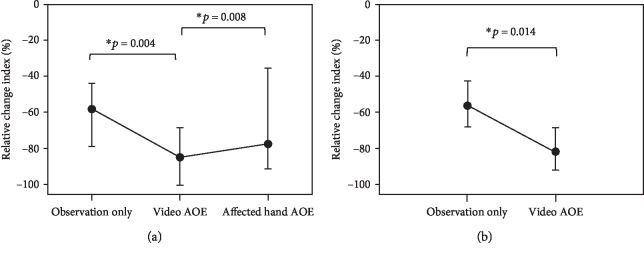
Comparisons of the relative change index of beta oscillatory activity under each condition of the stroke patients and healthy controls. (a) Stroke patients: the relative change index under the video AOE condition decreased significantly more than those under the observation condition and the affected hand AOE condition. (b) Healthy controls: the relative change index under the video AOE condition was significantly lower than that under the observation condition. Data are presented as the median ± IQR values. Note: AOE = action observation and execution.

Figure 3 shows the grand-averaged time-frequency analysis under each condition of the 2 groups of stroke patients and healthy controls. Within the group of stroke patients, there were significant differences in the relative change indices among the 3 conditions (*p* = 0.004). The post hoc analyses indicated significant differences between the observation only and video AOE conditions (*p* = 0.004) and between the video AOE and affected hand AOE conditions (*p* = 0.008). The relative change index of the beta oscillatory activity under the video AOE condition (median = ‐84.81, IQR = ‐100.49 to -68.33) was significantly lower than that under the observation only condition (median = ‐58.18, IQR = ‐78.81 to -44.06) and significantly lower than that under the condition of affected hand AOE (median = ‐77.39, IQR = ‐91.07 to -35.25) (Figure 4(a)). However, there was no significant difference between the observation only and affected hand AOE (*p* = 0.53) conditions in stroke patients.

In the healthy controls, the relative change index under the video AOE condition (median = ‐81.65, IQR = ‐91.91 to -68.67) was significantly lower than that under the observation only condition (median = ‐56.37, IQR = ‐68.07 to -42.78) (*p* = 0.014) (Figure 4(b)).

**Figure 5:**
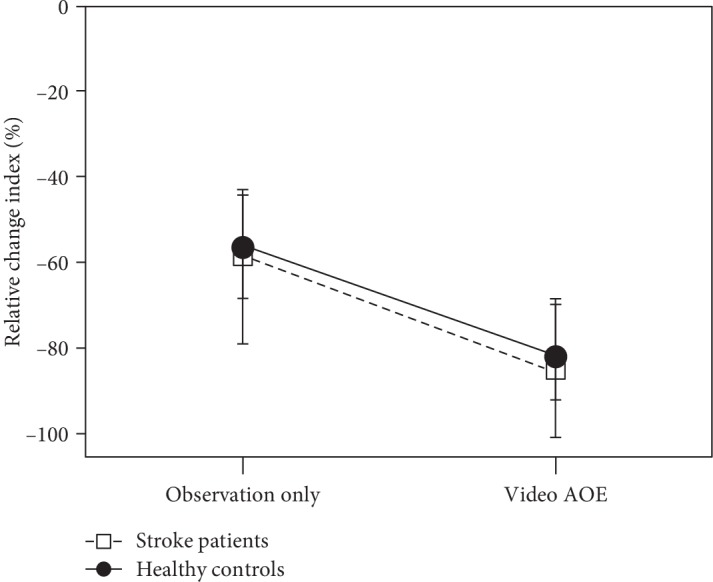
Comparisons of relative change indices under the conditions of observation only and video AOE between the stroke patients and healthy controls. No significant differences in relative change indices under the observation only and video AOE conditions were found between the 2 groups. Data are presented as the median ± IQR values. Note: AOE = action observation and execution.

Figure 5 summarizes the relative change indices under the observation only and video AOE conditions between the stroke patients and healthy controls. There were no significant differences in the relative change indices of the observation only (*p* = 0.70) and video AOE (*p* = 0.36) conditions between the 2 groups. There was a similar trend of a greater decrease in the relative change index under the video AOE condition than that under the observation only condition in the 2 groups.

In addition, to examine whether the time after stroke and Fugl-Meyer Assessment scores of the patients affected their ability to respond to the MEG experimental conditions, we analyzed the correlations between the 2 variables and the relative change index of beta oscillatory activity, respectively, for each condition in the stroke group. We found nonsignificant and low correlations between the time after stroke and the relative change index of beta oscillatory activity (*r* = 0.08 to 0.27, *p* = 0.25 to 0.74). Also found were nonsignificant and low correlations between the Fugl-Meyer Assessment score and the relative change index of beta oscillatory activity (*r* = ‐0.02 to -0.15, *p* = 0.54 to 0.93).

## 4. Discussion

Three main findings of this study are highlighted. First, we found that a significantly lower power of beta oscillatory activity was induced in stroke patients under the video AOE condition than that under the observation only and affected hand conditions. This finding indicated that more M1 cortical excitabilities of stroke patients could be activated and increased when the video of an action was simultaneously viewed and executed, providing more neurophysiological evidence to support the use of action observation therapy in stroke rehabilitation. Second, one particular new finding is that patients with stroke exhibited stronger M1 activations when watching a video of normal movement and executing the movement than when observing the abnormal movement of their own affected hands. This result indicates the benefits of observing normal and correct movements for motor learning in patients. Another main result was that the observation only and affected hand AOE conditions did not significantly induce different strengths of M1 activations in patients with stroke. This finding implies that, for patients with severe motor impairments, observing videos of actions might be an alternative strategy to watching the movements and executing them with the affected hand. These neurophysiological findings suggest that action observation therapy that combines observing videos of actions of healthy actors and executing the same actions might be a useful approach in stroke rehabilitation.

We found that action observation and execution induced greater M1 activities than did observation only in both groups of stroke patients and healthy controls. The human mirror neuron network can be activated during the learning of motor skills through observation of another individual performing a motor action as well as through execution of a similar action [6, 13]. A recent review also concluded that action observation activated the mirror neuron network and motor cortex, which may enhance motor learning in patients with stroke [18]. A previous study showed that congruent action observation with physical training promoted the formation of motor memory and led to positive effects of motor training after stroke [22]. Another study found neuroplastic changes induced by action observation and execution during the recovery course of stroke patients [26]. Based on the present and previous studies, observation of another healthy individual's movements in association with physical practice might enhance motor learning in stroke patients, supporting the potential application of action observation and execution in stroke rehabilitation.

The modulation of the M1 cortical excitability was significantly stronger in patients observing the normal movement patterns of a healthy actor in a video than in patients observing the abnormal patterns performed by their own affected hands (i.e., video AOE vs. affected hand AOE). In common and traditional clinical contexts, patients with stroke often watch the movement of their affected hand during rehabilitation training. However, the visual input and feedback of normal and correct movement patterns may improve motor relearning in stroke patients [44, 45]. The present study is the first to examine the differences between observing normal versus abnormal movements performed by another healthy person or by one's own affected hand in patients with stroke. A recent study conducted in healthy volunteers also showed that the M1 activity was stronger during the observation of normal movement than during the observation of abnormal, distorted movement [30]. These findings suggest that providing visual inputs of correct and normal movement patterns to patients may facilitate motor learning by inducing greater motor cortical activations. In clinics, for patients with voluntary movement, observing video clips of normal movements and executing the movements might thus be a good rehabilitation approach to relearn motor skills.

Our results also showed that the observation only and affected hand AOE conditions could induce similar degrees of neural activities of stroke patients. Although somewhat greater M1 neural activity was observed under the affected hand AOE condition than under the observation only condition, the activations did not significantly differ between the 2 conditions. One important implication of this finding is that observing a video of an action carefully but not needing to perform the movements physically could also induce M1 activations similar to those induced under the condition of observing and executing the action with the affected hand. Prior studies have indicated that during observation and imitation of an action, motor memory and motor performance can also be facilitated in stroke patients through the activation of neural networks similar to the areas activated by action execution [11, 24, 26, 46]. In addition, observing an action is viewed as a cognitive task which requires less physical effort [23]. Thus, for patients with severe motor deficits or no voluntary movement, observing videos of actions might be a promising alternative strategy to watching the movements and executing them with the affected hand in rehabilitation clinics.

Some limitations of this study warrant further consideration. First, the heterogeneity of the patients' characteristics may affect the generalization of the present results. Most of the stroke patients were male with moderate to mild motor deficits, and 14 participants had right hemisphere lesions (i.e., nondominant hand affected). However, there was no significant difference in the relative change indices of beta oscillatory activities between the patients with right and left hemisphere lesions in each experimental condition. In addition, most patients involved subcortical lesions in this study. Further study is suggested to examine if there is a differential effect of patients with cortical or subcortical stroke on action observation and execution. Second, the scale of this study was modest, for 20 patients and 20 healthy controls completed the experiment. Further larger-scale studies with more homogeneous patients are recommended. Third, although nonsignificant and low correlations were found between the 2 variables (time after stroke and Fugl-Meyer Assessment score) and the relative change index of beta oscillatory activity in this study, further investigation to identify which clinical characteristics of stroke patients might elicit more responses to action observation and execution would be worthwhile.

## 5. Conclusions

Our study results provide novel insights into the neural mechanisms underlying action observation and execution in patients with stroke and suggest targeted rehabilitation strategies for patients with different levels of motor impairment in clinics.

For patients with some voluntary movement, observing videos of movements and simultaneously practicing the movements seem a good strategy to induce greater activation in the primary motor cortex, rather than observing movements performed by the patients' affected hands, as commonly performed in clinics. In addition, the observation only and affected hand AOE induced similar degrees of motor cortical excitability, suggesting the potential benefits of observing videos of actions for motor learning in patients with severe motor deficits or those without voluntary movement. Our MEG findings could help guide the development of refined clinical protocols for action observation treatment for use in stroke rehabilitation.

## Figures and Tables

**Table 1:** Demographic and clinical characteristics of the participants with stroke.

No.	Gender	Age (years)	Duration (months)	Stroke type	Lesion location	Affected hand	FMA-UE
1	M	47	5	Hemorrhagic	Right putamen	L	45
2	M	39	2	Hemorrhagic	Right basal ganglia	L	42
3	F	57	9	Hemorrhagic	Left basal ganglia	R	30
4	M	55	2	Hemorrhagic	Left thalamus	R	58
5	M	38	2	Ischemic	Right corona radiata	L	60
6	M	42	4	Hemorrhagic	Right putamen	L	57
7	M	46	3	Ischemic	Right corona radiata	L	48
8	M	46	5	Ischemic	Right middle cerebral artery	L	60
9	M	34	4	Ischemic	Right middle cerebral artery	L	59
10	M	55	7	Ischemic	Left caudate head	R	56
11	M	62	11	Hemorrhagic	Right basal ganglia	L	58
12	M	48	11	Ischemic	Left internal capsule	R	51
13	M	62	6	Ischemic	Right corona radiata	L	51
14	M	38	4	Hemorrhagic	Left basal ganglia	R	37
15	M	63	9	Ischemic	Right corona radiata	L	41
16	M	49	8	Hemorrhagic	Right basal ganglia	L	53
17	M	50	2	Ischemic	Right periventricular area	L	58
18	M	47	4	Ischemic	Left corona radiata	R	58
19	M	37	1	Ischemic	Right basal ganglia, corona radiata, and fronto-temporo-parietal cortex	L	55
20	M	56	1	Hemorrhagic	Right putamen	L	60

Abbreviations: FMA-UE = upper-extremity subscale of Fugl-Meyer Assessment.

## Data Availability

The data used to support the findings of this study are available from the corresponding author upon request.
